# Effect of Vaccination against SARS-CoV-2 on Long COVID-19: A Narrative Review

**DOI:** 10.3390/life12122057

**Published:** 2022-12-08

**Authors:** Andreas G. Tofarides, Eirini Christaki, Haralampos Milionis, Georgios K. Nikolopoulos

**Affiliations:** 1 Department of Internal Medicine, Nicosia General Hospital, Nicosia 2029, Cyprus; 2 Department of Internal Medicine, Faculty of Medicine, School of Health Sciences, University of Ioannina, 451 10 Ioannina, Greece; 3 Medical School, University of Cyprus, Nicosia 2029, Cyprus

**Keywords:** SARS-CoV-2 vaccination, long COVID-19, post-COVID-19 condition, SARS-CoV-2, SARS-CoV-2 vaccines, variant, immunity, post-COVID-19 sequelae

## Abstract

Vaccines against SARS-CoV-2 have saved millions of lives and played an important role in containing the COVID-19 pandemic. Vaccination against SARS-CoV-2 is also associated with reduced disease severity and, perhaps, with COVID-19 symptom burden. In this narrative review, we present, in a clinically relevant question-and-answer manner, the evidence regarding the association between vaccination against SARS-CoV-2 and long COVID-19. We discuss how the mechanism of action of vaccines could interplay with the pathophysiology of post-COVID-19 condition. Furthermore, we describe how specific factors, such as the number of vaccine doses and the type of SARS-CoV-2 variants, may affect post-COVID-19 condition. We also discuss the role of timing for vaccination in relation to the onset of long COVID-19 symptoms, as it seems to affect the frequency and severity of the condition. Additionally, we describe the potential modifying effect of age, as well as the association of type and level of immune response with long COVID-19. We also describe how system-specific long COVID-19 sequelae, namely neurocognitive-psychologic symptoms and cardiovascular pathology, could be altered by vaccination. Last, we address the question of whether seasonal influenza vaccination has a meaningful impact on the frequency of long COVID-19.

## 1. Introduction

The pandemic of SARS-CoV-2, the causative agent of COVID-19, started in December 2019 and has led to more than 600 million confirmed infections and over 15 million deaths [1,2,3]. COVID-19 entails a wide and variable clinical spectrum, from asymptomatic infection and mild disease to pneumonia with respiratory failure and systemic critical illness with multi-organ failure [4,5].

Apart from the management of acute COVID-19, a new clinical syndrome has emerged. An increasing number of people continue to experience symptoms that cannot be explained by alternative diagnoses weeks or months after an initial mild or severe SARS-CoV-2 infection. The condition is referred to as “long COVID-19”, “post-acute sequelae of SARS-CoV-2 infection (PASC)”, “chronic COVID-19”, “post-COVID syndrome”, “post-acute COVID-19”, or “post-COVID-19 condition” [6,7,8,9,10,11]. A standardized clinical case definition of post-COVID-19 condition was developed by the Word Health Organization (WHO) through a Delphi consensus process and was published on 6 October 2021: “Post COVID-19 condition occurs in individuals with a history of probable or confirmed SARS-CoV-2 infection, usually 3 months from the onset of COVID-19 with symptoms that last for at least 2 months and cannot be explained by an alternative diagnosis. Common symptoms include fatigue, shortness of breath, cognitive dysfunction, but also others, which generally have an impact on everyday functioning. Symptoms may be new onset following initial recovery from an acute COVID-19 episode or persist from the initial illness. Symptoms may also fluctuate or relapse over time.” [12]. Similar syndromes have been described in the aftermath of other infections. These include viral pathogens such as the Epstein-Barr virus (EBV), Retroviruses, Human Herpesvirus 6 (HHV-6), Enteroviruses, and the Ross river virus [13,14] as well as bacterial infections such as Lyme disease, a tick-borne illness, caused by one of the three pathogenic species of the spirochete *Borrelia* (most commonly *B.burgdorferi, B afzeli, and B.garinil*) [14,15].

According to the last technical report from the European Centre for Disease Prevention and Control (ECDC), which was based on a systematic review and meta-analysis [16,17,18,19,20], the five most common symptoms of post-COVID-19 condition are fatigue, shortness of breath, depression, headache, and dizziness. Each of these five symptoms is more prevalent in hospitalized patients. More specifically, the prevalence of any post-COVID-19 condition was estimated at 50.6% (95% Confidence Interval (Cl): 41.1–60.2%, moderate certainty) among cohorts referring to community patients, at 66.5% (95% Cl: 56.0–76.3%, moderate certainty) in cohorts referring to hospitalized patients, and at 73.8% (95% Cl: 62.3–83.9%, low certainty) among cohorts of patients admitted to an Intensive Care Unit (ICU). In another attempt to estimate the global burden of long COVID-19, investigators pooled data from cohort and database studies and found that in 1.2 million individuals with symptomatic SARS-CoV-2 infection in 2020–2021, 6.2% (95% uncertainty interval: 2.4–13.3%) reported at least 1 of the 3 long COVID-19 symptom clusters (persistent fatigue with bodily pain or mood swings, cognitive problems, or ongoing respiratory problems) [21]. Furthermore, recent evidence suggests that the prevalence of persistent symptoms may vary depending on the COVID-19 variant [22,23,24].

Vaccines against SARS-CoV-2 have saved millions of lives and played a pivotal role in curbing the COVID-19 pandemic [25,26,27,28]. Vaccination against SARS-CoV-2 is also associated with reduced disease severity and, perhaps, symptom burden of COVID-19 [29,30,31,32,33,34,35]. The initial SARS-CoV-2 vaccine clinical trials involving messenger RNA (mRNA) [BNT 162b2(Pfizer-BioNTech), mRNA-1273(Moderna)] or non-mRNA vaccines, such as vaccines based on a replication-incompetent chimpanzee adenovirus vector [ChAdox1 nCoV-19/AZD 1222 (University of Oxford, AstraZeneca, and the Serum Institute of India), Ad26.CoV.2S (Jcovden—previously Janssen)], showed very high efficacy, up to 90–91% for individuals more than 65 years, 95% for those 16 years or older and 100% for individuals 12 to 15 years [36,37,38]. Despite their similarly increased effectiveness against any or severe infection, as evidenced by real-world data [35], the protection from COVID-19 vaccines, especially from infection and symptomatic disease, diminishes over time. This is probably the result of waning immunity over time (four months after vaccination) and immune evasion by novel circulating SARS-CoV-2 variants (for example Omicron variants BA.1, BA.2, BA.2.12.1, BA.4, and BA.5) [29]. However, more recently it was shown that despite decreased vaccine effectiveness in preventing SARS-CoV-2 infection, the risk of severe disease remains dramatically lower in vaccinated adults, even with the emergence of new variants [39]. For example, in one study [40] in the United States (US) that included 10 million adults, the effectiveness of three vaccines (BNT 162b2, mRNA-1273, Ad26.CoV2.S) was 54–70% against infection, 86–90% against hospitalization, and 90–93% against death 5 months after the booster dose among participants who had completed a primary vaccines series. Consequently, these studies established that vaccines continue to provide a good level of protection against severe disease, hospitalization, and death. Vaccines against COVID-19 have proved to be not only effective but also safe. The evidence on safety is quite robust and based on a huge amount of real-world data. For example, according to the US Centers for Disease Control and Prevention (CDC) [41], more than 636 million doses of COVID-19 vaccines have been given in the US from 14 December 2020 until 27 October 2022. These COVID-19 vaccines were safe, and adverse effects attributed to them were very rare. The most important adverse events included a small risk of myocarditis associated with mRNA vaccines [42] and a very rare syndrome of thrombosis with thrombocytopenia [43] and the Guillain-Barre Syndrome (GBS) [44,45], observed among people who received adenoviral vector-based vaccines.

More evidence is also being accumulated on the association between COVID-19 vaccination and post-COVID-19 condition [46,47,48]. However, since our understanding of the pathophysiologic mechanisms and natural course of long COVID-19 are still not clear, several questions arise on the implications of vaccination on post-COVID-19 condition. For example, whether prior vaccination reduces the impact of post-COVID-19 condition, if any vaccination or vaccination timing, before or after infection, affects long COVID-19 occurrence and severity, whether age influences the effectiveness of vaccination or if vaccination plays a role in post-COVID-19 neurocognitive and cardiovascular sequelae. Hence, the main objective of this narrative review is to discuss the current evidence on the role of primary and booster vaccination in the occurrence, severity, and duration of post-COVID-19 condition [46,47,48].

## 2. Vaccination against SARS-CoV-2 and Post-COVID-19 Condition (Long COVID-19)

### 2.1. Question #1: What Is the Impact of SARS-CoV-2 Vaccination on the Pathophysiology of Acute and Long COVID-19?

#### 2.1.1. Question #1a: Can the Impact of SARS-CoV-2 Vaccination Be Explained by Similarities in the Pathophysiologic Mechanisms of Acute and Long COVID-19?

**Figure 1:**
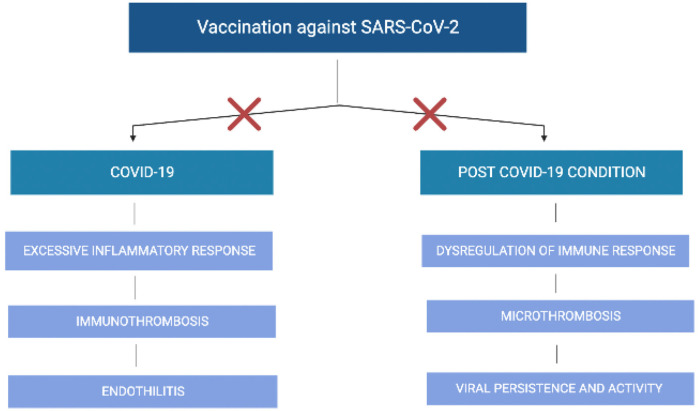
Potential effect of vaccination against SARS-CoV-2 on the disruption of the pathophysiologic mechanisms that lead to COVID-19 and post-COVID-19 condition.

The host receptor for SARS-CoV-2 cell entry is the angiotensin-converting enzyme-2 (ACE-2). SARS-CoV-2 binding is mediated by the receptor-binding domain (RBD) of its spike protein, and cellular entry is facilitated by the cellular serine protease TMPRSS2. Cell entry and subsequent viral replication cause a range of clinical manifestations, from mild symptoms to severe pneumonia and respiratory failure [49]. The relative abundance of the ACE2 receptor, which is present in the heart, lung, pancreas, kidney, gastrointestinal tract, blood vessels, liver, and brain may explain the multiple organs affected by COVID-19 and the variability of clinical presentation between individuals. On the other hand, the biological underpinnings responsible for the development of long COVID-19 symptoms are not yet elucidated. One hypothesis for the pathophysiology of long COVID-19 is that it represents a continuation of the acute disease endotype, namely direct and persistent viral activity resulting in cell damage, endothelitis, and immune-mediated microthrombosis [50]. Dysregulation of the immune response, sustained activation of a hyperinflammatory condition, and autoimmune-mediated organ dysfunction related to the development of antigen-antibody complexes or mast cell activation are other plausible mechanistic pathways that could explain long-COVID-19 symptoms [51]. As both acute and chronic COVID-19 may share similar biologic disturbances, it is reasonable to expect that vaccination against SARS-CoV-2 could inhibit the progression of initial infection to a post-COVID-19 condition by interrupting the same pathways (Figure 1).

#### 2.1.2. Question #1b: Can the Mechanism of Action of mRNA Vaccines Attenuate Post-COVID-19 Condition?

The mechanism of action of mRNA vaccines may interfere with the pathophysiology mechanisms of acute and long COVID-19. After administration of mRNA vaccines, mRNA-LNPs (lipid nanoparticle) or locally produced antigens are received from antigen-presenting cells (APCs), such as professional APCs (dendritic cells, macrophages, and B-cells) and atypical APCs (mast cells, basophils, eosinophils, and ILC 3s) [52]. In the lymph nodes, APCs interact with CD4 and CD8-T lymphocytes. CD8-T lymphocytes can induce the formation of cytotoxic T lymphocytes, which respond with the production of interferon-γ (IFN-γ), interleukin-2 (IL-2), Tumor Necrosis Factor (TNF), perforin, and granzymes, contributing to the direct killing of the pathogen. In one study [53], a significant increase in SARS-CoV-2 specific CD8-T cells (measured by IFN-γ production from CD8-T cells following interaction with vaccine antigens) was found after vaccination with mRNA vaccines. Furthermore, CD4-T cells can differentiate into Th1 cells or T follicular helper (Tfh) cells. Tfh cells are crucial regulators of the humoral immune response and are found in significant numbers in lymph nodes, spleen, and blood, after immunization with mRNA vaccines [54]. In addition, it seems that after the administration of SARS-CoV-2 vaccines, CD4-T cells polarize towards a Th1 response, while Tfh cells are characterized by the production of both Th1 (IFN-γ) and Th2 (IL-4) cytokines [55]. It seems that interleukins produced by CD4-T cells and CD8-T cells due to vaccination are in a strict equilibrium. Relevant to the pathophysiologic pathways of long COVID-19, a study by Vijayakumar et al. [56] showed proteomic and immunological abnormalities in the lungs of patients with persistent respiratory symptoms 3 and 6 months after acute infection. More specifically, lung bronchoalveolar lavage (BAL) showed activation of CD8-T cytotoxic cells and elevated levels of some proteins, such as granzyme K, granzyme A, and perforin, and this was associated with epithelial injury, apoptosis, necrosis, and tissue repair. Consequently, CD4 T-cells and CD8 T-cells play an important role in the immune response after vaccination and SARS-CoV-2 infection and perhaps in the pathophysiology of long COVID-19 syndrome. However, more research is definitely needed in order to determine how cellular immune responses from different vaccines could variably affect post-COVID-19 condition.

### 2.2. Question #2: Does Vaccination Reduce the Occurrence, Severity or Duration of Post-COVID-19 Condition?

#### 2.2.1. 2a: Vaccination and Occurrence of Post-COVID-19 Condition According to the Number of Doses and the Circulating Variant

Several investigators have tried to explore whether SARS-CoV-2 vaccination could also reduce the symptom burden of long COVID-19. A retrospective study from Israel [57] that included 951 infected and 2437 uninfected individuals examined whether vaccination was associated with the reported incidence of long-term symptoms after SARS-CoV-2 infection. Vaccination coverage among the infected participants was 67%. The most common symptoms were fatigue (22%), headache (20%), weakness (13%), and persistent muscle pain (10%). Those who received two vaccine doses were significantly less likely than the unvaccinated to mention any of these symptoms by 64%, 54%, 57%, and 68%, respectively. From these results, it seems that vaccination against COVID-19 with at least two doses reduces the most common long COVID-19 symptoms. Additionally, in a recent study in the US of 16,091 adults, 14.7% continued to experience COVID-19 symptoms two months after the infection, whereas, prior to acute illness, complete but not partial vaccination was associated with diminished risk for long COVID-19 [Odds Ratio (OR): 0.72; 95% Confidence Interval (CI): 0.60–0.86] [24]. Another prospective cohort studied 2560 patients with mild COVID-19 [47]. Symptoms were present in 16% of those vaccinated with three doses, in 17.4% of those with two doses, and in 30% of those with only one dose, while in unvaccinated individuals the prevalence was much higher (42.8%). In another observational study [22], which included over 97,000 vaccinated individuals in the United Kingdom (UK), breakthrough infection with the Omicron variant was associated with a lower risk of developing persistent symptoms compared with the Delta variant infection [4.5% versus (vs.) 10.8%]. Omicron cases were less likely to have long COVID-19, irrespective of vaccine timing, with an OR ranging from 0.24 (95% CI: 0.20–0.32) to 0.50 (95% CI: 0.43–0.59). Furthermore, another case-control study from the UK examined 8400 people who received either one dose (*n* = 6030) or two doses (*n* = 2370) of a COVID-19 vaccine, prior to acute SARS-CoV-2 infection. Participants who were vaccinated with two doses were about half as likely as the unvaccinated participants to have post-COVID-19 condition, lasting for 28 days or longer (OR: 0.51, 95% CI: 0.32–0.82) [46]. Overall, it seems that vaccination reduces the occurrence, severity, and duration of post-COVID-19 condition. However, because studies have different designs, vaccine types, and timings of symptom assessment, results may not be entirely comparable.

#### 2.2.2. 2b: Level and Type of Serological Response in Previously Unvaccinated Individuals vs. Vaccinated Individuals and Post-COVID-19 Condition

It is not clear whether only the presence or also the type of immunity (from vaccination or infection) or the titer of antibodies against SARS-CoV-2 influence the intensity of post-COVID-19 condition. It has been suggested that the persistence of high serological response induced by natural infection but not by vaccination may play a role in long COVID-19. A prospective study [58] investigated post-COVID-19 condition, 6 and 12 months after disease onset, in adults who had been diagnosed with COVID-19 between March and May 2020. Of the 479 individuals (52.6% female, mean age 53 years) who were interviewed 13.5 months after acute infection, 132 (27.5%) were vaccinated and 347 were unvaccinated. Patients were considered vaccinated if they had received the vaccine at least two weeks prior to the interview. The presence of non-RBD SARS-CoV-2 IgG induced by natural infection showed a significant association with long-COVID-19 (OR: 1.35, 95% CI: 1.11–1.64). Moreover, median non-RBD SARS-CoV-2 IgG titers after one year were significantly higher (*p* = 0.009) in patients with long COVID-19 symptoms [22 kAU/L, Interquartile Range (IQR): 9.7–37.2] than in patients without symptoms (14.1 kAU/L, IQR: 5.4–31.3). On the other hand, the presence of RBD SARS-CoV-2 IgG was not significantly associated with the occurrence of post-COVID-19 condition (OR: 1.36, 95% CI: 0.62–3.00). Furthermore, RBD SARS-CoV-2 IgG titers did not differ between patients with long COVID-19 symptoms and those without symptoms, despite natural infection. So far, there are no sufficient data to support a safe prediction of the duration of vaccine or natural infection protection against post-COVID-19 condition.

#### 2.2.3. 2c: How Does Timing of Vaccination Affect Post-COVID-19 Condition?

A retrospective study in the US examined individuals with long COVID-19 who were vaccinated during randomized periods after acute SARS-CoV-2 infection. The results showed that the earlier individuals receive their first dose of a COVID-19 vaccine, the less likely they are to develop long COVID-19 symptoms between 12 and 20 weeks after COVID-19 diagnosis [59]. Furthermore, a study by Al-Aly et al. [48] indicated that patients who have breakthrough infections after vaccination, compared with unvaccinated patients, had a lower risk of death and long COVID-19 including cardiovascular, mental health, coagulation, hematologic, musculoskeletal, and neurologic disorders between 1 and 6 months after the infection. The overall protection of the vaccine against long COVID-19 syndrome was estimated at approximately 15%, conferring thus only partial protection from post-COVID-19 condition.

Summarizing the evidence, most published studies conclude that previous vaccination with two or three doses reduces long COVID-19 symptoms. Vaccination status and especially the number of doses and the timing before or after infection are both important.

### 2.3. Question #3: Does Later Vaccination Reduce the Impact of Post-COVID-19 Condition in Unvaccinated Patients?

Vaccination might be beneficial even after the development of long COVID-19, as shown in unvaccinated individuals who reported symptoms months after the acute phase of COVID-19 and who were vaccinated despite being symptomatic. A prospective cohort study by Arnold et al. [60] examined the effect of vaccination (with BNT162b2-Pfizer-BioNTech vaccine or ChAdOx1 nCoV-19/AZD1222-AstraZeneca) on long COVID-19 symptoms eight months after the infection in unvaccinated participants who were previously hospitalized with COVID-19. Participants who were vaccinated, even while being symptomatic, experienced improvement in their symptoms compared with unvaccinated participants. Similar findings were reported by a prospective cohort of Ayoubkhani et al. [61], who showed that vaccination with primary and booster doses improves symptoms in participants with post-COVID-19 condition who were vaccinated while having long COVID-19 symptoms, irrespective of the vaccine type. Furthermore, the study by Strain et al [62] showed the effectiveness of vaccination in long COVID-19, even in previously unvaccinated people, since participants who were vaccinated with the mRNA-1273 vaccine were the most likely to report improvement and the least likely to report deterioration of their symptoms.

### 2.4. Question #4: Does Age Influence the Effectiveness of Vaccination οn Post-COVID-19 Condition?

The prospective cohort study by Kuodi et al. [57] showed that participants with 2 or 3 vaccine doses were 54% to 83% less likely than the unvaccinated to report seven of the ten most commonly reported symptoms. Vaccination effectiveness against reported symptoms related to long COVID-19 was the highest for older participants (>60 years) and the lowest for younger participants (19 to 35 years). A case-control study by Antoneli et al. [46] involving 6030 participants showed that fully vaccinated participants were about half as likely as unvaccinated participants to have symptoms lasting ≥28 days (OR: 0.51, 95% CI: 0.32–0.82); moreover, fully vaccinated adults aged 18 to 59 years were much less likely to have symptoms lasting ≥28 days than unvaccinated adults. Therefore, given the paucity of studies to answer this question, more research is needed in order to determine whether patient age is associated with vaccine effectiveness in preventing post-COVID-19 symptoms.

### 2.5. Question #5: Can Vaccination Prevent Neurocognitive and Psychologic Disorders in Post-COVID-19?

Neurocognitive and psychological disorders, post-traumatic stress disorder, impaired memory, poor concentration, anxiety, and depression have been described in post-COVID-19 condition and affect quality of life. More commonly, the symptom of “brain fog” is reported by patients with long COVID-19 [63,64,65,66]. Several mechanisms have been under investigation to explain the neurocognitive and psychological sequelae of COVID-19. Chronic inflammation, microangiopathy, loss of oligodendrocytes, autoimmunity, T-cell mediated neuroinflammation, and natural killer cell dysfunction have been associated with brain fog [67,68,69]. In addition, it seems that patients who are infected with COVID-19 have greater loss of gray matter, greater tissue damage, and a greater decrease in whole brain volume [70]. Vaccination against SARS-CoV-2 may be an important intervention to avoid the long-term effects of COVID-19, such as brain fog, in the case of a breakthrough infection. First of all, there is no association between neurocognitive or psychological disorders and vaccination [60,61,71,72]. Moreover, data suggest that persistent symptoms are less likely to worsen and could perhaps even improve following the administration of the SARS-CoV-2 vaccine. This was shown clearly in one study of 163 patients who had severe long COVID-19 symptoms at eight months and who subsequently received the BNT162b2 -Pfizer-BioNTech or ChAdOx1 nCoV-19/AZD1222 vaccine. One month after vaccination, symptoms that existed prior to vaccination either improved or remained unchanged in the majority of patients, whereas only 5% reported worsening of symptoms [60,61,71,72].

### 2.6. Question #6: Can Vaccination Prevent Cardiovascular Complications of Post-COVID-19?

Long-term cardiovascular sequelae of post-COVID-19 are well described. Some common symptoms include chest pain, postural tachycardia syndrome (POTS), arrhythmia, and exertional dyspnea. Furthermore, more severe complications such as myocarditis, stress cardiomyopathy (takotsumbo), endothelitis, injury due to hypoxia, cardiac microvascular dysfunction, and epicardial coronary disease (plaque rupture or demand ischemia) have been reported [73,74]. However, the contribution of these mechanisms to myocardial damage is not clearly defined.

Among patients who had been infected with SARS-CoV-2, the risk of cardiovascular disease (CVD) is perhaps elevated [75]. Notably, prior myocardial injury or structural heart disease is not a prerequisite for the presence of symptoms defying the classical definitions of viral myocarditis. Subclinical diffuse cardiovascular inflammation is increasingly recognized as a risk factor in chronic autoimmune systemic conditions [76]. Furthermore, in a prospective study that recorded the annual incidence of CVD among 5.8 million US veterans, patients with a history of COVID-19 had an increased risk of death or major adverse cardiovascular events (myocardial infarction, stroke, heart failure, arrhythmias) in contrast with a group of patients who did not have COVID-19 [75]. Also, patients hospitalized with more severe COVID-19 were more likely to develop cardiovascular disease when compared with patients without severe COVID-19.

COVID-19 may represent a temporary risk factor for cardiovascular disease and thus patient follow-up is necessary. Nevertheless, vaccination, in order to prevent COVID-19-associated CVD, sounds promising. A retrospective US cohort study by Al-Aly et al. [48] examined if vaccination for COVID-19 before infection was associated with post-acute sequelae of COVID-19, including cardiovascular disorders, in adults who had a positive test for COVID-19. In that study, vaccinated cases (*n* = 16,035, mean age of 67 years, 91% male) were matched to unvaccinated controls (*n* = 48,536, mean age of 56 years, 86% male). The analysis showed that vaccinated participants were less likely to have at least 1 post-acute sequelae of COVID-19 at 6 months compared with unvaccinated individuals. However, more evidence is necessary in order to determine if vaccination is a protective factor against cardiovascular complications. 

### 2.7. Question #7: Does Influenza Vaccination Reduce the Prevalence of Long COVID-19 Syndrome?

COVID-19 and influenza are different respiratory tract infections but with similar clinical features during the acute phase of the disease. Their long-term effects, however, are somewhat different. Vaccination against influenza and its potentially serious complications is very important for people who are at higher risk of developing serious complications. At this time, it is not clear how and if influenza vaccination reduces the prevalence of long COVID-19.

SARS-CoV-2 virus and influenza type A virus represent two highly transmissible airborne pathogens with pandemic capabilities. The two viruses are similar in their ability to infect human airways and may co-exist, resulting in significant morbidity and mortality [77,78,79,80,81]. These viruses use different host receptors for cell entry: SARS-CoV-2 binds to ACE2 with its spike protein, while influenza A recognizes receptors with saccharides terminating in sialic acid-α2,6-galactose with its hemagglutinin protein. SARS-CoV-2 and influenza A seem to infect alveolar type II cells [82,83,84]. From the beginning of the pandemic, the question of whether previous vaccination against influenza A affected infection due to SARS-CoV-2 was plausible and, therefore, it can be posed again regarding its effects on long COVID-19. In one multivariate regression model from Israel [85], among 715,164 members of a health insurance organization, the odds for SARS-CoV-2 infection among individuals vaccinated for influenza in 2018–2019 or 2019–2020 were significantly lower (20–25%) than those in non-vaccinated individuals. Although further evidence is needed, influenza vaccination is highly recommended to prevent influenza and morbidity when SARS-CoV-2 and influenza viruses are co-circulating [86,87]. 

A summary of the answers to the questions mentioned above is given in Table 1.

## 3. Conclusions

Apart from the acute management of patients with COVID-19, a novel challenge has emerged. Approximately one in five patients who were infected with SARS-CoV-2 experience long-term symptoms, which cannot be attributed to another disease. This entity, defined as long COVID-19 or post-COVID-19 condition, has been recognized and research on its pathogenesis, pathophysiology, clinical course, and long-term effects is expected to shed light οn similar post-infectious syndromes such as those associated with EBV, HHV- 6, enteroviruses, and Lyme disease, which have been understudied. Until now, long COVID-19 remains a syndrome without specific treatment. Thus, the preventive and therapeutic challenge of post-COVID-19 condition is of urgent clinical priority. Recently, it was shown that treatment with nirmatrelvir, when indicated in the acute phase of COVID-19, was associated with a reduced risk of post-acute sequelae, regardless of vaccination status and history of prior infection [88]. Additionally, and more importantly, based on the existing evidence, it seems that there is a protective effect of vaccination on long COVID-19. This protective effect can be influenced by the number of doses, the circulating variant, the timing of vaccination before or after infection, and, possibly, age. Moreover, vaccination seems to prevent neurocognitive-psychological disorders and cardiovascular complications. Last, it is not clear whether other vaccines, such as the influenza vaccine, reduce the burden of long COVID-19, but routine vaccination is highly recommended to prevent influenza and morbidity when SARS-CoV-2 and influenza viruses are co-circulating. Finally, given the significant burden of the disease, countries should immediately take action against long COVID-19, implementing guidelines for post-COVID-19 diagnosis and management, creating patient registries, as well as improving access to long-term follow-up for better monitoring with specific medical facilities to enhance our understanding of this condition.

## Figures and Tables

**Table 1:** Association between vaccination and long COVID-19: Evidence from the literature.

Vaccination and Long COVID-19	Association	References
Pathophysiology of acute and long COVID-19 and the impact of SARS-CoV-2 vaccination	Acute and long-COVID-19 share common pathophysiologic pathways. Vaccination could attenuate immune responses leading to chronic symptoms	[46,47,48,49,50,51,52,53]
Number of vaccine doses and post-COVID-19 condition	Two doses or complete vaccination is likely more protective than one dose	[24,46,47,57]
Circulating variants and post-COVID-19 condition	Inconclusive/inadequate evidence	[22]
Type and level of serological response and post-COVID-19 condition	Persistence of high serological response induced by natural infection but not by vaccination may play a role in long COVID-19	[58]
Timing of vaccination and post-COVID-19 condition	Earlier vaccination, before infection, may be more protective	[48,59]
Vaccination during long-COVID-19 symptoms in previously unvaccinated adults	Improvement of symptoms is likely	[57,58,59,60,61,62]
Age and effectiveness of vaccination in relation to post-COVID-19 condition	Effectiveness is likely higher in older adults; however, more data is needed	[46,57]
Vaccination and neurocognitive and cardiovascular post-COVID-19 sequalae	Improvement/protection is likely; however, more data is needed	[48,57,60,61,62,63,64,65,66,67,68,69,70,71,72,73,74,75,76]

## References

[1] worldometers.info/coronavirus.

[2] World Health Organization (2020). Director-General’s remarks at the Media Briefing on 2019-nCoV on 11 February 2020. http://www.who.int/dg/speeches/detail/who-director-general-s-remarks-at-the-media-briefing-on-2019-ncov-on-11-february-2020.

[3] Wang H., Paulson K.R., Pease S.A., Watson S., Comfort H., Zheng P., Aravkin A.Y., Bisignano C., Barber R.M., Alam T. (2022). Estimating excess mortality due to the COVID-19 pandemic: A systematic analysis of COVID-19-related mortality, 2020–21. Lancet.

[4] Kamal M., Omirah M.A., Hussein A., Saeed H. (2021). Assessment and characterisation of post-COVID-19 manifestations. Int. J. Clin. Pract..

[5] Jiang F., Deng L., Zhang L., Cai Y., Cheung C.W., Xia Z. (2020). Review of the Clinical Characteristics of Coronavirus Disease 2019 (COVID-19). J. Gen. Intern. Med..

[6] Carfì A., Bernabei R., Landi F. (2020). Persistent Symptoms in Patients After Acute COVID-19. JAMA.

[7] Barman M.P., Rahman T., Bora K., Borgohain C. (2020). COVID-19 pandemic and its recovery time of patients in India: A pilot study. Diabetes Metab. Syndr. Clin. Res. Rev..

[8] Prescott H.C., Angus D.C. (2018). Enhancing Recovery from Sepsis. JAMA.

[9] Xiong Q., Xu M., Li J., Liu Y., Zhalng J., Xu Y., Dong W. (2021). Clinical sequelae of COVID-19 survivors in Wuhan, China: A single-centre longitudinal study. Clin. Microbiol. Infect..

[10] Goërtz Y.M.J., Van Herck M., Delbressine J.M., Vaes A.W., Meys R., Machado F.V.C., Houben-Wilke S., Burtin C., Posthuma R., Franssen F.M.E. (2020). Franssen Persistent symptoms 3 months after a SARS-CoV-2 infection: The post-COVID-19 syndrome?. ERJ Open Res..

[11] Evaluating and Caring for Patients with Post-COVID Conditions: Interim Guidance. https://www.cdc.gov/coronavirus/2019-ncov/hcp/clinical-care/post-covid-index.html.

[12] Word Organization Health (WHO) (2021). A Clinical Case Definition of Post COVID-19 Condition by a Delphi Consensus, 6 October 2021; WHO. https://www.who.int/publications/i/item/WHO-2019-nCoV-Post_COVID-19_condition-Clinical_case_definition-2021.1.

[13] Oakes B., Hoagland-Henefield M., Komaroff A.L., Erickson J.L., Huber B.T. (2013). Human Endogenous Retrovirus-K18 Superantigen Expression and Human Herpesvirus-6 and Human Herpesvirus-7 Viral Loads in Chronic Fatigue Patients. Clin. Infect. Dis..

[14] Hickie I., Davenport T., Wakefield D., Vollmer-Conna U., Cameron B., Vernon S.D., Reeves W.C., Lloyd A. (2006). Post-infective and chronic fatigue syndromes precipitated by viral and non-viral pathogens: Prospective cohort study. BMJ.

[15] Coyle P.K., Krupp L.B., Doscher C., Amin K. (1994). Borrelia burgdorferi Reactivity in Patients with Severe Persistent Fatigue Who Are from a Region in Which Lyme Disease Is Endemic. Clin. Infect. Dis..

[16] European Centre for Disease Prevention and Control (2022). Prevalence of Post COVID-19 Condition Symptoms: A Systematic Review and Meta-Analysis of Cohort Study Data Stratified by Recruitment Setting.

[17] Alkodaymi M.S., Omrani O.A., Fawzy N.A., Shaar B.A., Almamlouk R., Riaz M., Obeidat M., Obeidat Y., Gerberi D., Taha R.M. (2022). Prevalence of post-acute COVID-19 syndrome symptoms at different follow-up periods: A systematic review and meta-analysis. Clin. Microbiol. Infect..

[18] Malik P., Patel K., Pinto C., Jaiswal R., Tirupathi R., Pillai S., Patel U. (2022). Post-acute COVID-19 syndrome (PCS) and health-related quality of life (HRQoL)—A systematic review and meta-analysis. J. Med. Virol..

[19] Michelen M., Manoharan L., Elkheir N., Cheng V., Dagens A., Hastie C., O’Hara M., Suett J., Dahmash D., Bugaeva P. (2021). Characterising long COVID: A living systematic review. BMJ Glob. Health..

[20] Moreno-Pérez O., Merino E., Leon-Ramirez J.-M., Andres M., Ramos J.M., Arenas-Jiménez J., Asensio S., Sanchez R., Ruiz-Torregrosa P., Galan I. (2021). Post-acute COVID-19 syndrome. Incidence and risk factors: A Mediterranean cohort study. J. Infect..

[21] Hanson S.W., Abbafati C., Aerts J.G., Al-Aly Z., Ashbaugh C., Ballouz T., Blyuss O., Bobkova P., Bonsel G., Global Burden of Disease Long COVID Collaborators (2022). Estimated Global Proportions of Individuals with Persistent Fatigue, Cognitive, and Respiratory Symptom Clusters Following Symptomatic COVID-19 in 2020 and 2021. JAMA.

[22] Antonelli M., Pujol J.C., Spector T.D., Ourselin S., Steves C.J. (2022). Risk of long COVID associated with delta versus omicron variants of SARS-CoV-2. Lancet.

[23] Arjun M.C., Singh A.K., Roy P., Ravichandran M., Mandal S., Pal D., Das K., Gajjala A., Venkateshan M., Mishra B. (2022). Long COVID following Omicron wave in Eastern India—A retrospective cohort study. J. Med Virol..

[24] Perlis R.H., Santillana M., Ognyanova K., Safarpour A., Trujillo K.L., Simonson M.D., Green J., Quintana A., Druckman J., Baum M.A. (2022). Prevalence and Correlates of Long COVID Symptoms among US Adults. JAMA Netw. Open.

[25] Watson O.J., Barnsley G., Toor J., Hogan A.B., Winskill P., Ghani A.C. (2022). Global impact of the first year of COVID-19 vaccination: A mathematical modelling study. Lancet Infect. Dis..

[26] Feikin D.R., Higdon M.M., Abu-Raddad L.J., Andrews N., Araos R., Goldberg Y., Groome M.J., Huppert A., O’Brien K.L., Smith P.G. (2022). Duration of effectiveness of vaccines against SARS-CoV-2 infection and COVID-19 disease: Results of a systematic review and meta-regression. Lancet.

[27] Stephenson J. (2021). US COVID-19 Vaccination Efforts May Have Prevented More than 1 Million Deaths, 10 Million Hospitalizations. JAMA Health. Forum.

[28] Steele M.K., Couture A., Reed C., Iuliano D., Whitaker M., Fast H., Hall A.J., MacNeil A., Cadwell B., Marks K.J. (2022). Estimated Number of COVID-19 Infections, Hospitalizations, and Deaths Prevented among Vaccinated Persons in the US, December 2020 to September 2021. JAMA Netw. Open.

[29] Havers F.P., Pham H., Taylor C.A., Whitaker M., Patel K., Anglin O., Kambhampati A.K., Milucky J., Zell E., Moline H.L. (2022). COVID-19-Associated Hospitalizations among Vaccinated and Unvaccinated Adults 18 Years or Older in 13 US States, January 2021 to April 2022. JAMA Intern. Med..

[30] McNamara L.A., Wiegand R.E., Burke R.M., Sharma A.J., Sheppard M., Adjemian J., Ahmad F.B., Anderson R.N., Barbour K.E., Binder A.M. (2021). Estimating the early impact of the US COVID-19 vaccination programme on COVID-19 cases, emergency department visits, hospital admissions, and deaths among adults aged 65 years and older: An ecological analysis of national surveillance data. Lancet.

[31] Joseph G., Barnes J., Azziz-Baumgartner E., Arvay M., Fry A., Hall A., Kutty P., MacNeil A., Donald L.C., Reynolds S. (2022). Association of mRNA Vaccination with Clinical and Virologic Features of COVID-19 among US Essential and Frontline Workers. JAMA.

[32] Andrews N., Tessier E., Stowe J., Gower C., Kirsebom F., Simmons R., Gallagher E., Thelwall S., Groves N., Dabrera G. (2022). Duration of Protection against Mild and Severe Disease by Covid-19 Vaccines. N. Engl. J. Med..

[33] Laake I., Skodvin S.N., Blix K., Caspersen I.H., Gjessing H.K., Juvet L.K., Magnus P., Mjaaland S., Robertson A.H., Starrfelt J. (2022). Effectiveness of mRNA booster vaccination against mild, moderate, and severe COVID-19 caused by the Omicron variant in a large, population-based, Norwegian cohort. J. Infect. Dis..

[34] Barouch D.H. (2022). Covid-19 Vaccines—Immunity, Variants, Boosters. N. Engl. J. Med..

[35] Ferdinands J.M., Rao S., Dixon B.E., Mitchell P.K., DeSilva M.B., Irving S.A., Lewis N., Natarajan K., Stenehjem E., Grannis S.J. (2022). Waning 2-Dose and 3-Dose Effectiveness of mRNA Vaccines Against COVID-19–Associated Emergency Department and Urgent Care Encounters and Hospitalizations among Adults during Periods of Delta and Omicron Variant Predominance—VISION Network, 10 States, August 2021–January 2022. MMWR Morb. Mortal. Wkly. Rep..

[36] Polack F.P., Thomas S.J., Kitchin N., Absalon J., Gurtman A., Lockhart S., Perez J.L., Pérez Marc G., Moreira E.D., Zerbini C. (2020). Safety and efficacy of the BNT162b2 mRNA COVID-19 vaccine. N. Engl. J. Med..

[37] Frenck R.W., Klein N.P., Kitchin N., Gurtman A., Absalon J., Lockhart S., Perez J.L., Walter E.B., Senders S., Bailey R. (2021). Safety, Immunogenicity, and Efficacy of the BNT162b2 Covid-19 Vaccine in Adolescents. N. Engl. J. Med..

[38] Thomas S.J., Moreira E.D., Kitchin N., Absalon J., Gurtman A., Lockhart S., Perez J.L., Pérez Marc G., Polack F.P., Zerbini C. (2021). Safety and Efficacy of the BNT162b2 mRNA Covid-19 Vaccine through 6 Months. N. Engl. J. Med..

[39] Ioannou B.G.N., Locke M.E.R., O’Hare A.M., Bohnert A.S., Boyko E.J., Hynes M.D.M., Berry K. (2022). COVID-19 Vaccination Effectiveness Against Infection or Death in a National, U.S. Health Care System. Ann. Intern. Med..

[40] Lin D.-Y., Gu Y., Xu Y., Wheeler B., Young H., Sunny S.K., Moore Z., Zeng D. (2022). Association of Primary and Booster Vaccination and Prior Infection With SARS-CoV-2 Infection and Severe COVID-19 Outcomes. JAMA.

[41] Centres for Disease Control and Prevention (CDC) Safety of COVID-19 Vaccines. https://www.cdc.gov/coronavirus/2019-ncov/vaccines/safety/safety-of-vaccines.html.

[42] Gargano J.W., Wallace M., Hadler S.C., Langley G., Su J.R., Oster M.E., Broder K.R., Gee J., Weintraub E., Shimabukuro T. (2021). Use of mRNA COVID-19 Vaccine After Reports of Myocarditis among Vaccine Recipients: Update from the Advisory Committee on Immunization Practices—United States, June 2021. MMWR. Morb. Mortal. Wkly. Rep..

[43] Schultz N.H., Sørvoll I.H., Michelsen A.E., Munthe L.A., Lund-Johansen F., Ahlen M.T., Wiedmann M., Aamodt A.-H., Skattør T.H., Tjønnfjord G.E. (2021). Thrombosis and Thrombocytopenia after ChAdOx1 nCoV-19 Vaccination. N. Engl. J. Med..

[44] McDonnell E.P., Altomare N.J., Parekh Y.H., Gowda R.C., Parikh P.D., Lazar M.H., Blaser M.J. (2020). COVID-19 as a Trigger of Recurrent Guillain–Barré Syndrome. Pathogens.

[45] Abu-Rumeileh S., Abdelhak A., Foschi M., Tumani H., Otto M. (2021). Guillain–Barré syndrome spectrum associated with COVID-19: An up-to-date systematic review of 73 cases. J. Neurol..

[46] Antonelli M., Penfold R.S., Merino J., Sudre C.H., Molteni E., Berry S., Canas L.S., Graham M.S., Klaser K., Modat M. (2022). Risk factors and disease profile of post-vaccination SARS-CoV-2 infection in UK users of the COVID Symptom Study app: A prospective, community-based, nested, case-control study. Lancet Infect. Dis..

[47] Azzolini E., Levi R., Sarti R., Pozzi C., Mollura M., Mantovani A., Rescigno M. (2022). Association between BNT162b2 Vaccination and Long COVID after Infections Not Requiring Hospitalization in Health Care Workers. JAMA.

[48] Al-Aly Z., Bowe B., Xie Y. (2022). Long COVID after breakthrough SARS-CoV-2 infection. Nat. Med..

[49] Wiersinga W.J., Rhodes A., Cheng A.C., Peacock S.J., Prescott H.C. (2020). Pathophysiology, Transmission, Diagnosis, and Treatment of Coronavirus Disease 2019 (COVID-19). JAMA.

[50] Garg M., Maralakunte M., Garg S., Dhooria S., Sehgal I., Bhalla A.S., Vijayvergiya R., Grover S., Bhatia V., Jagia P. (2021). The Conundrum of ‘Long-COVID-19’: A Narrative Review. Int. J. Gen. Med..

[51] Weinstock L.B., Brook J.B., Walters A.S., Goris A., Afrin L.B., Molderings G.J. (2021). Mast cell activation symptoms are prevalent in Long-COVID. Int. J. Infect. Dis..

[52] Bettini E., Locci M. (2021). SARS-CoV-2 mRNA Vaccines: Immunological Mechanism and Beyond. Vaccines.

[53] Lu J., Lu G., Tan S., Xia J., Xiong H., Yu X., Qi Q., Yu X., Li L., Yu H. (2020). A COVID-19 mRNA vaccine encoding SARS-CoV-2 virus-like particles induces a strong antiviral-like immune response in mice. Cell Res..

[54] Tai W., Zhang X., Drelich A., Shi J., Hsu J.C., Luchsinger L., Hillyer C.D., Tseng C.-T.K., Jiang S., Du L. (2020). A novel receptor-binding domain (RBD)-based mRNA vaccine against SARS-CoV-2. Cell Res..

[55] Crotty S. (2019). T Follicular Helper Cell Biology: A Decade of Discovery and Diseases. Immunity.

[56] Vijayakumar B., Boustani K., Ogger P.P., Papadaki A., Tonkin J., Orton C.M., Ghai P., Suveizdyte K., Hewitt R.J., Desai S.R. (2022). Immuno-proteomic profiling reveals aberrant immune cell regulation in the airways of individuals with ongoing post-COVID-19 respiratory disease. Immunity.

[57] Kuodi P., Gorelik Y., Zayyad H., Wertheim O., Wiegler K.B., Abu Jabal K., Dror A.A., Nazzal S., Glikman D., Edelstein M. (2022). Association between BNT162b2 vaccination and reported incidence of post-COVID-19 symptoms: Cross-sectional study 2020-21, Israel. NPJ Vaccines.

[58] Peghin M., De Martino M., Palese A., Gerussi V., Bontempo G., Graziano E., Visintini E., D’Elia D., Dellai F., Marrella F. (2022). Post–COVID-19 syndrome and humoral response association after 1 year in vaccinated and unvaccinated patients. Clin. Microbiol. Infect..

[59] Simon M.A., Luginbuhl R.D., Parker R. (2021). Reduced Incidence of Long-COVID Symptoms Related to Administration of COVID-19 Vaccines Both before COVID-19 Diagnosis and Up to 12 Weeks after. medRxiv.

[60] Arnold D.T., Milne A., Samms E., Stadon L., Maskell N.A., Hamilton F.W. (2021). Symptoms After COVID-19 Vaccination in Patients with Persistent Symptoms After Acute Infection: A Case Series. Ann. Intern. Med..

[61] Ayoubkhani D., Bermingham C., Pouwels K.B., Glickman M., Nafilyan V., Zaccardi F., Khunti K., Alwan N.A., Walker A.S. (2022). Trajectory of long covid symptoms after covid-19 vaccination: Community based cohort study. BMJ.

[62] Strain W.D., Sherwood O., Banerjee A., van der Togt A., Hishmeh L., Rossman J. (2022). The Impact of COVID Vaccination on Symptoms of Long COVID: An International Survey of People with Lived Experience of Long COVID. Vaccines.

[63] Greenhalgh T., Knight M., A’Court C., Buxton M., Husain L. (2020). Management of post-acute covid-19 in primary care. BMJ.

[64] National Institutes of Health (NIH) (2021). NIH Launches New Initiative to Study ‘Long COVID. https://www.nih.gov/about-nih/who-we-are/nih-director/statements/nih-launches-new-initiative-study-long-covid.

[65] National Institute for Health and Care Excellence (UK) (2022). COVID-19 Rapid Guideline: Managing the Long-Term Effects of COVID-19. https://pubmed.ncbi.nlm.nih.gov/33555768/.

[66] Alwan N.A., Johnson L. (2021). Defining long COVID: Going back to the start. Med.

[67] British Society for Immunology (2020). Long-Term Immunological Health Consequences of COVID-19 (British Society for Immunology, 2020). https://www.immunology.org/sites/default/fles/BSI_Briefng_Note_August_2020_FINAL.pdf.

[68] di Toro A., Bozzani A., Tavazzi G., Urtis M., Giuliani L., Pizzoccheri R., Aliberti F., Fergnani V., Arbustini E. (2021). Long COVID: Long-term effects?. Eur. Heart J. Suppl..

[69] Zhang L., Richards A., Barrasa M.I., Hughes S.H., Young R.A., Jaenisch R. (2021). Reverse-transcribed SARS-CoV-2 RNA can integrate into the genome of cultured human cells and can be expressed in patient-derived tissues. Proc. Natl. Acad. Sci. USA.

[70] Abbasi J. (2022). Even Mild COVID-19 May Change the Brain. JAMA.

[71] Whittaker H.R., Gulea C., Koteci A., Kallis C., Morgan A.D., Iwundu C., Weeks M., Gupta R., Quint J.K. (2021). GP consultation rates for sequelae after acute covid-19 in patients managed in the community or hospital in the UK: Population based study. BMJ.

[72] Wisnivesky J.P., Govindarajulu U., Bagiella E., Goswami R., Kale M., Campbell K.N., Meliambro K., Chen Z., Aberg J.A., Lin J.J. (2022). Association of Vaccination with the Persistence of Post-COVID Symptoms. J. Gen. Intern. Med..

[73] Raman B., Bluemke D.A., Lüscher T.F., Neubauer S. (2022). Long COVID: Post-acute sequelae of COVID-19 with a cardiovascular focus. Eur. Heart J..

[74] Raj S.R., Arnold A.C., Barboi A., Claydon V.E., Limberg J.K., Lucci V.-E.M., Numan M., Peltier A., Snapper H., Vernino S. (2021). Long-COVID postural tachycardia syndrome: An American Autonomic Society statement. Clin. Auton. Res..

[75] Xie Y., Xu E., Bowe B., Al-Aly Z. (2022). Long-term cardiovascular outcomes of COVID-19. Nat. Med..

[76] Puntmann V.O., Martin S., Shchendrygina A., Hoffmann J., Ka M.M., Giokoglu E., Vanchin B., Holm N., Karyou A., Laux G.S. (2022). Long-term cardiac pathology in individuals with mild initial COVID-19 illness. Nat. Med..

[77] Jacek C., Karolina S., Orzeł A., Frączek M., Tomasz Z. (2021). Comparison of the clinical differences between COVID-19, SARS, influenza, and the common cold: A systematic literature review. Adv. Clin. Exp. Med..

[78] Flerlage T., Boyd D.F., Meliopoulos V., Thomas P.G., Schultz-Cherry S. (2021). Influenza virus and SARS-CoV-2: Pathogenesis and host responses in the respiratory tract. Nat. Rev. Microbiol..

[79] Musher D.M. (2021). Bacterial Coinfection in COVID-19 and Influenza Pneumonia. Am. J. Respir. Crit. Care Med..

[80] Bai Y., Tao X. (2021). Comparison of COVID-19 and influenza characteristics. J. Zhejiang Univ. Sci. B.

[81] Chotpitayasunondh T., Fischer T.K., Heraud J., Hurt A.C., Monto A.S., Osterhaus A., Shu Y., Tam J.S. (2021). Influenza and COVID-19: What does co-existence mean?. Influenza Other Respir. Viruses.

[82] Hoffmann M., Kleine-Weber H., Schroeder S., Krüger N., Herrler T., Erichsen S., Schiergens T.S., Herrler G., Wu N.-H., Nitsche A. (2020). SARS-CoV-2 Cell Entry Depends on ACE2 and TMPRSS2 and Is Blocked by a Clinically Proven Protease Inhibitor. Cell.

[83] Weis W., Brown J.H., Cusack S., Paulson J.C., Skehel J.J., Wiley D.C. (1988). Structure of the influenza virus haemagglutinin complexed with its receptor, sialic acid. Nature.

[84] Traylor Z.P., Aeffner F., Davis I.C. (2013). Influenza A H1N1 induces declines in alveolar gas exchange in mice consistent with rapid post-infection progression from acute lung injury to ARDS. Influenza Other Respir. Viruses.

[85] Green I., Ashkenazi S., Merzon E., Vinker S., Golan-Cohen A. (2021). The association of previous influenza vaccination and coronavirus disease-2019. Hum. Vaccines Immunother..

[86] Pilkington E.H., Suys E.J., Trevaskis N.L., Wheatley A.K., Zukancic D., Algarni A., Al-Wassiti H., Davis T.P., Pouton C.W., Kent S.J. (2021). From influenza to COVID-19: Lipid nanoparticle mRNA vaccines at the frontiers of infectious diseases. Acta Biomater..

[87] Conlon A., Ashur C., Washer L., Eagle K.A., Hofmann Bowman M.A. (2021). Impact of the influenza vaccine on COVID-19 infection rates and severity. Am. J. Infect. Control.

[88] Xie Y., Al-Aly Z. (2022). Nirmatrelvir and the Risk of Post-Acute Sequelae of COVID-19. medRxiv.

